# 5-methoxytryptophan: an arsenal against vascular injury and inflammation

**DOI:** 10.1186/s12929-020-00671-w

**Published:** 2020-07-07

**Authors:** Kenneth K. Wu, Cheng-Chin Kuo, Shaw-Fang Yet, Chii-Ming Lee, Jun-Yang Liou

**Affiliations:** 1 Institute of Cellular and System Medicine, National Health Research Institutes, 35 Keyan Road, Zhunan Town, Miaoli County, 35053 Taiwan; 2 College of Life Sciences, National Tsing-Hua University, Hsinchu, Taiwan; 3 School of Medicine, China Medical University, Taichung, Taiwan; 4 College of Medicine, National Taiwan University, Taipei, Taiwan

**Keywords:** 5-methoxytryptophan, Tryptophan hydroxylase-1, Hydroxyindole O-methyltransferase, Intimal hyperplasia, Endothelial barrier function, Smooth muscle cell migration and proliferation, Macrophage activation, Sepsis, Chronic renal failure, Heart failure

## Abstract

5-methoxytryptophan (5-MTP) is an endothelial factor with anti-inflammatory properties. It is synthesized from L-tryptophan via two enzymatic steps: tryptophan hydroxylase-1 (TPH-1) and hydroxyindole O-methyltransferase. Lipopolysaccharide (LPS) and pro-inflammatory cytokines suppress endothelial 5-MTP production by inhibiting TPH-1 expression. 5-MTP protects endothelial barrier function and promotes endothelial repair, while it blocks vascular smooth muscle cell migration and proliferation by inhibiting p38 MAPK activation. 5-MTP controls macrophage transmigration and activation by inhibiting p38 MAPK and NF-κB activation. 5-MTP administration attenuates arterial intimal hyperplasia, defends against systemic inflammation and prevents renal fibrosis in relevant murine models. Serum 5-MTP level is depressed in human sepsis as well as in mice with sepsis-like disorder. It is reduced in chronic kidney disease and acute myocardial infarction in humans. The reported data suggest that serum 5-MTP may be a theranostic biomarker. In summary, 5-MTP represents a new class of tryptophan metabolite which defends against inflammation and inflammation-mediated tissue damage and fibrosis. It may be a valuable lead compound for developing new drugs to treat complex human inflammatory disorders.

## Background

Vascular endothelium is lined with a single layer of cobblestone like endothelial cells (ECs) which interface with circulating blood [1]. It is endowed with well-coordinated molecular machinery to maintain a tight cellular junction and barrier function. Furthermore, ECs secrete a number of factors to protect endothelium, regulate vascular tone and prevent blood cell adhesion and aggregation. Thus, endothelium provides a natural barrier to maintain vascular and blood homeostasis. Under stress conditions, however, endothelium alters its protective functions and switches to a pro-inflammatory and pro-thrombotic phenotype.

Under physiological conditions, ECs produce small molecules, notably prostacyclin (also known as Prostaglandins I_2_, PGI_2_) and nitric oxide (NO) to protect endothelial integrity and defend against blood cell adhesion and aggregation [2]. PGI_2_ was discovered in 1970’s as an endothelial factor with anti-platelet aggregation action and vaso-dilating property. It is synthesized from arachidonic acid, an unsaturated fatty acid derived from membrane phospholipids, via two enzymatic steps, i.e. cyclooxygenase (COX also known as prostaglandin H synthase) and prostacyclin synthase. PGI_2_ exerts multiple effects on vascular functions. It inhibits platelet aggregation, relaxes vascular smooth muscle cells (SMCs) and protects endothelial integrity. Reduced PGI_2_ production increases risk of arterial thrombosis, cardiovascular diseases and pulmonary hypertension. NO was discovered to be an endothelial factor in the 1990’s. It relaxes vascular SMCs and is considered as a major factor in regulating vascular tone. 5-methoxytryptophan (5-MTP) was recently identified as a new endothelial factor with vasoprotective and anti-inflammatory actions [3]. Thus, 5-MTP joins PGI_2_ and NO as key molecules in protecting arterial integrity and function.

5-MTP was discovered as a COX-2 suppressing factor derived from proliferative fibroblasts [4]. Its suppression of cancer cell COX-2 expression is accompanied by inhibition of cancer cell migration, epithelial mesenchymal transition and cancer metastasis [4, 5]. It was subsequently reported that vascular cells are the major source of 5-MTP [3]. 5-MTP has been regarded as a "new member of endothelial arsenal against inflammation [6]. This review will focus on endothelial 5-MTP biosynthesis, its vasoprotective functions and mechanisms of actions.

### Biosynthesis and secretion of 5-MTP in ECs

Investigations so far reveal that 5-MTP synthesis is restricted to human fibroblasts, vascular ECs and SMCs, bronchial and renal epithelial cells [3]. Its synthetic pathway has been characterized in fibroblasts and ECs. 5-MTP is synthesized from intracellular tryptophan via two enzymatic steps: tryptophan hydroxylase (TPH) converts L-tryptophan to 5-hydroxytryptophan (5-HTP) and hydroxyindole O-methyltransferase (HIOMT) converts 5-HTP to 5-MTP [4]. Of the two TPH isoforms [7, 8] only TPH-1 isoform is detected in fibroblasts and ECs [3, 9]. Silencing of TPH-1 with siRNA leads to a marked reduction of 5-MTP production. Thus, 5-MTP synthesis is catalyzed by TPH-1 isoform. It is not entirely certain as to which isoform of HIOMT catalyzes 5-MTP synthesis. Recent published data suggest that HIOMT298 isoform is the active isoform in 5-MTP synthesis [10]. HIOMT is known to catalyze the final step of melatonin synthesis in pineal and retinal cells. As it catalyzes the conversion of N-acetylserotonin to N-acetyl-5-methoxytryptamine (melatonin), HIOMT is commonly known as ASMT (acetylserotonin methyltransferase). ASMT was reported to be encoded by a single gene [11]. Three mRNA isoforms were reported in pineal cells: isoform 373 codes for a 373 amino acid (aa) ASMT protein while isoform 345 and isoform 298 code for 345 aa and 298 aa proteins, respectively [11, 12]. Structure-function analysis based on X-ray crystallography indicates that isoform 345 is active in catalyzing melatonin synthesis while isoforms 373 and 298 are inactive [13]. ASMT345 is the major isoform expressed in pineal cells and retinoblastoma cell line Y-79. By contrast, only isoform 298 mRNA is detected in human ECs and fibroblasts [10]. Cancer cells express a very low level of HIOMT298 and are deficient in 5-MTP synthesis. Stable transfection of HIOMT298 in A549 lung cancer cells restores 5-MTP synthesis [10]. These results suggest that HIOMT298 is the active isoform catalyzing 5-MTP synthesis [10]. The data are inconclusive. Further studies are needed to provide direct evidence that HIOMT298 proteins are catalytically active in 5-MTP synthesis.

Immunofluorescent staining of human umbilical vein ECs (HUVECs) with 5-MTP antibodies reveals cytosolic staining with endoplasmic reticulum (ER) staining pattern [3]. Pretreatment of ECs with ER to Golgi transport inhibitors results in shutdown of 5-MTP release into the extracellular milieu [3]. These results suggest that 5-MTP is secreted into extracellular milieu via Golgi vesicular transport. Constitutive endothelial production and release of 5-MTP contributes to a high level of 5-MTP in human circulating blood [3]. Circulating 5-MTP serves as an autocoid to protect blood vessels and control inflammation.

### 5-MTP supplement restores protection of endothelial barrier function

At physiological conditions, ECs maintain a well-regulated barrier which prevents blood cells, macromolecules, electrolytes and other small molecules from leaking into the extravascular space. The barrier function is supported by dynamic junction protein complexes among which vascular endothelial (VE) cadherins are the anchor [14, 15]. Vascular endothelial growth factor (VEGF), lipopolysaccharide (LPS) and pro-inflammatory cytokines disrupt the barrier by degrading VE-cadherin [16]. Disruption of endothelial barrier is a critical step in inflammation.

Pro-inflammatory mediators suppress TPH-1 expression in ECs thereby reducing 5-MTP production [3, 17]. Addition of 5-MTP to HUVECs rescues endothelium from LPS- and cytokine-induced barrier disruption by preventing degradation of VE-cadherin, thus maintaining endothelial tight junction [17]. 5-MTP blocks LPS- and cytokine-induced leakage of FITC-labeled dextran or Evans blue dye through vascular wall [3].

p38 mitogen-activated protein kinase (MAPK) is a crucial signaling pathway via which LPS and pro-inflammatory cytokines perturb endothelial barrier function [18, 19]. Furthermore, it mediates leukocyte adhesion and transmigration [19]. Inhibition of LPS- and cytokine-induced p38 MAPK activation by SB202190 attenuates VE-cadherin degradation thereby preserving barrier function [17]. 5-MTP blocks LPS- and cytokine-induced p38 MAPK activation but not ERK activation [17]. It is likely that 5-MTP protects endothelial barrier function through inhibition of p38 MAPK signaling pathway.

### 5-MTP defends against injury-induced endothelial denudation and intimal hyperplasia

It was reported in murine models that following mechanical or ischemic injury, ECs are damaged and detached resulting in denudation [20–23]. In response to injury, vascular SMCs migrate to intimal layer and proliferate. Furthermore, they release pro-inflammatory cytokines and collagen, resulting in intimal hyperplasia [20, 21]. 5-MTP administration to the murine vascular injury models results in attenuation of denudation and intimal hyperplasia [22, 23]. 5-MTP reduces endothelial loss by two possible mechanisms. First, it prevents oxidant-induced endothelial apoptosis. Our unpublished data show that 5-MTP protects HUVECs from H_2_O_2_-induced cell death. 5-MTP was reported to prevent H_2_O_2_-induced cardiomyocyte death [24]. Second, 5-MTP may restore re-endothelialization driven by VEGF. VEGF promotes endothelium repair by enhancing EC migration and proliferation via VEGF receptor 2 (VEGFR-2) [25, 26]. Pro-inflammatory cytokines which are accumulated locally interfere with VEGFR 2 phosphorylation and down-stream signaling, thereby blocking the action of VEGF [26]. 5-MTP may rescue VEGF-driven endothelial growth by blocking the effect of pro-inflammatory cytokines.

**Fig. 1:**
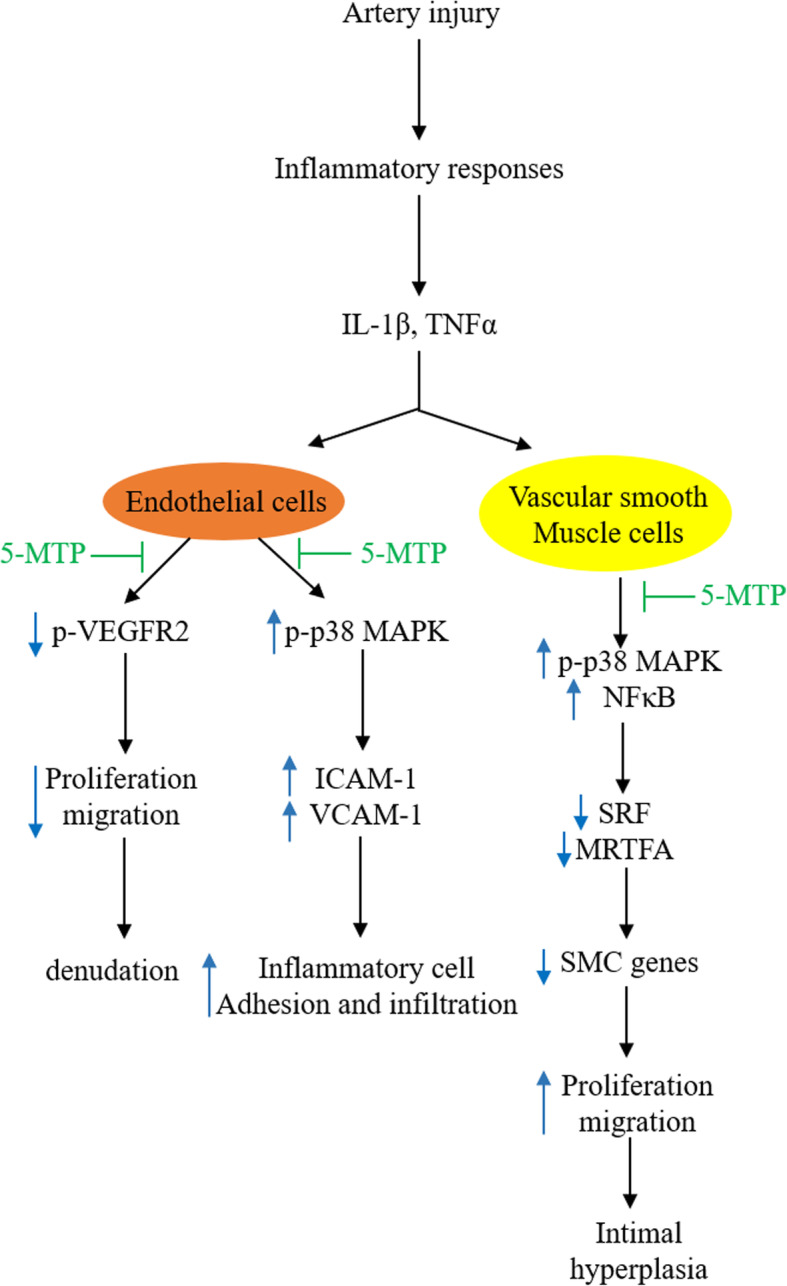
Illustrated summary of the vasoprotective actions of 5-MTP. 5-MTP restores EC migration and proliferation while it inhibits cytokine-induced expression of EC adhesion molecules and SMC migration and proliferation. It preserves SRF and MRTFA, thereby attenuating SMC phenotypic switch by blocking p38 MAPK and NF-κB activation

5-MTP attenuates intimal hyperplasia probably by inhibiting cytokine-induced vascular SMC migration and phenotypic switch. Interleukin-1β (IL-1β) and tumor necrosis factor α (TNFα) accumulation at the intimal region play an important role in intimal hyperplasia [27–29]. 5-MTP selectively inhibits cytokine-induced SMC migration and proliferation without an effect on VEGF-induced SMC migration and proliferation [23]. Expression of vascular SMC contractile proteins depends on binding of serum response factor (SRF) and its co-factor myocardin-related transcription factor-A (MRTF-A) to the promoter region of SMC marker genes, such as *SM-α actin* [30]. TNFα down-regulates expression of SRF and MRTF-A thereby suppressing SM-α actin expression [30]. As 5-MTP rescues SRF and MRTF as effectively as p38 MAPK inhibitors, it is likely that 5-MTP prevents cytokine-induced vascular SMC phenotypic switch through inactivation of p38 MAPK. Taken together, the reported results suggest that 5-MTP defends against injury-induced intimal hyperplasia by countering cytokine-induced SMC migration and phenotypic switch (Fig. 1).

IL-1β and TNFα activate p38 MAPK via a cascade of signaling pathways. Following binding to their respective receptors, they signal via distinct signaling pathways but eventually the signals converge at transforming growth factor β1-activated kinase-1 (TAK1) which occupies a pivotal position in activation of IκB kinase (IKK) and mitogen-activated protein kinase kinases (MKKs) [31, 32]. MKKs phosphorylate MAPKs, i.e. p38 MAPK, ERK and JNK which in turn activate transactivators notably CREB, C/EBP, NF-κB and AP-1. IKK, on the other hand phosphorylates IκB resulting in NF-κB activation [33–35]. By blocking p38 MAPK, 5-MTP inhibits NF-κB and an array of transactivators important in mediating SMC phenotypic switch. It has been reported that SRF and MRTF-A expressions in vascular SMCs are down-regulated by cytokines via p38 MAPK and NF-κB activation [36].

It is important to note that in vascular injury models, 5-MTP exerts an opposite effect on vascular SMC vs. EC migration and proliferation. 5-MTP promotes EC migration and proliferation while it blocks SMC migration and proliferation. The mechanism is unclear and requires further investigation.

### 5-MTP targets macrophages for control of systemic inflammation

Monocytes and macrophages are one of the key players in mediating systemic inflammation. Bacterial endotoxins such as LPS activate monocytes and macrophages resulting in production and release of multiple pro-inflammatory cytokines and chemokines and expression of pro-inflammatory enzymes such as COX-2 and inducible nitric oxide synthase (iNOS). Collectively, cytokines, chemokines and the enzyme products, such as prostaglandin E_2_ and NO induce severe inflammatory responses and tissue damages [37–39]. In humans, endotoxemia from bacterial infections often causes life threatening sepsis. An important feature of severe sepsis is a surge of blood cytokine levels, akin to the arrival of a storm (surge of cytokines was called “cytokine storm”). Systemic rise of proinflammatory cytokines, prostaglandins and reactive oxygen and nitrogen molecules causes widespread tissue damage and multi-organ failure. 5-MTP was reported to alleviate septic organ failure and mortality by targeting macrophages. 5-MTP inhibits LPS-induced expression of IL-1β, TNFα and interleukin-6 (IL-6) in murine peritoneal macrophages and Raw 264.7 cells at the transcriptional level [3]. It inhibits LPS-induced COX-2 transcription by suppressing NF-κB activation [3]. 5-MTP was reported to inhibit binding of multiple transactivators including NF-κB, c-Jun, C/EBPβ and CREB/ATF to the proximal region of COX-2 promoter in human fibroblasts [40]. It is likely that 5-MTP blocks COX-2, IL-1β, TNFα and IL-6 transcription by a common mechanism involving simultaneous inhibition of activation of multiple inflammation-mediated transactivators.

**Fig. 2:**
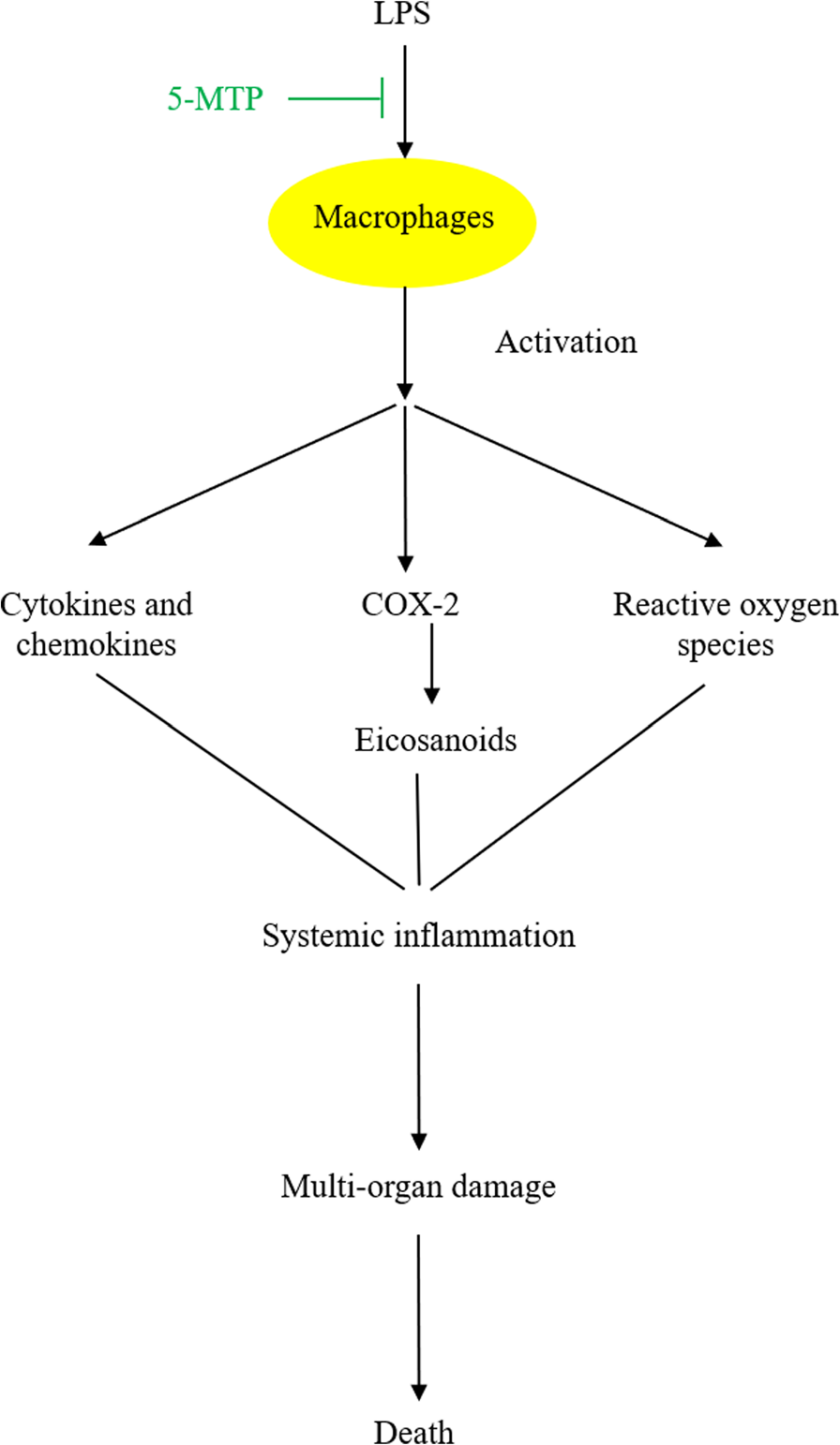
5-MTP defends against systemic inflammation. 5-MTP blocks LPS-induced macrophage activation and consequently inhibits release of macrophage-derived cytokines, eicosanoids and reactive oxygen species

5-MTP was reported to inhibit LPS-induced p300 histone acetyltransferase (HAT) activity [3]. p300 HAT acetylates histone and alters chromatin structure to facilitate transactivator binding [41]. Furthermore, it acetylates a large number of transactivators including p65 subunit of NF-κB, c-Jun, C/EBPβ and CREB to enhance their binding to inflammatory genes [42, 43]. Thus, p300 HAT occupies a pivotal position in inflammation. By inhibiting p300 HAT, 5-MTP re-enforces its inhibition of pro-inflammatory gene expressions. Figure 2 illustrates the transcriptional mechanism by which 5-MTP inhibits inflammatory gene expression.

### 5-MTP blocking macrophage activity by inhibiting p38 MAPK

p38 MAPK is known to be a major pathway via which LPS activates macrophages and induces systemic inflammation [44, 45]. Pretreatment of macrophages with 5-MTP inhibits LPS-induced p38 MAPK activation. 5-MTP was reported to inhibit LPS-induced p38 MAPK by interfering binding of phosphor-p38 to peroxiredoxins (Prdx). Prdx belong to peroxidase family which regulate H_2_O_2_ level and signaling [46]. They are ubiquitous and elevated in cancer cells [46]. A recent report suggests that Prdx-1 promotes pancreatic cancer growth by binding to phospho-p38 MAPK and sustaining p38 MAPK activation [47]. In LPS-treated macrophages, binding of p-p38 to Prdx-1 is augmented. Pretreatment of macrophages with 5-MTP abrogates the enhancing effect of LPS on p-p38 through inhibition of binding of p-p38 to Prdx-1 [3]. This is potentially a mechanism by which 5-MTP dampens p38 MAPK activation.

### 5-MTP ameliorates sepsis-like disorder in murine models

Intravenous injection of LPS in mice generates a sepsis-like systemic inflammatory disorder with elevation of serum cytokines and chemokines, multi-organ inflammatory cell infiltration and a high mortality. Macrophages in this model are activated and play a key role in inducing systemic inflammation. Intraperitoneal administration of 5-MTP to LPS-induced sepsis model results in suppression of macrophage activation and cytokine expression accompanied by reduction of serum cytokines (IL-6, IL-1β, TNFα and IFNγ) and chemokines (CXCL-1, MCP-1, Rantes and Eotaxin) [3]. Administration of 5-MTP to another sepsis model i.e. cecum ligation and perforation model also reduces serum IL-1β and IL-6 to the basal level. Thus, 5-MTP is effective in controlling macrophages activation and “cytokine storm” in vivo. 5-MTP attenuates lung inflammation in LPS sepsis model as evidenced by reduced inflammatory cell infiltration, COX-2 and iNOS expression as well as NF-κB and p300HAT activation in lung tissues [3]. Improvement of lung damage is accompanied by an increase in survival [3]. As summarized in Fig. 2, 5-MTP blocks LPS-induced classic activation of macrophages thereby suppressing the expression and release of pro-inflammatory cytokines, chemokines, eicosanoids and reactive oxygen species. Consequently, 5-MTP attenuates tissue damages by multiple inflammatory mediators and reduces mortality. These results suggest that 5-MTP could be a useful lead compound for developing new classes of therapeutic agents against systemic inflammation.

### 5-MTP ameliorates renal inflammation and fibrosis

Unilateral urethral obstruction (UUO) causes renal cell injury, interstitial inflammation and fibrosis [48]. Administration of 5-MTP to a murine UUO model was reported to attenuate inflammation, tissue damage and fibrosis which are accompanied by reduced COX-2, and MCP-1 expression and NF-κB activation [49]. These findings are consistent with the observations that 5-MTP is effective in controlling inflammatory responses thereby attenuating tissue damage, fibrosis and functional defects. Inflammation is a crucial determinant of outcomes following acute myocardial infarction (MI) and ischemic stroke. It will be important to evaluate the effects of 5-MTP on post-MI heart failure and post-stroke brain damage.

### Blood 5-MTP level as a biomarker of inflammation-mediated disorders

Several studies provide data to suggest that serum 5-MTP may be a biomarker for early detection and severity assessment of inflammation-mediated diseases. In a study of sepsis patients, mean serum 5-MTP level in 50 patients with sepsis was significantly lower than that of 30 healthy subjects [3]. Analysis of serum 5-MTP as a diagnostic test of sepsis by AU ROC (area under the receiver operating characteristic) curve reveals that serum 5-MTP is highly sensitive and specific for diagnosing severe sepsis [3]. In the case of chronic renal failure, serum 5-MTP was identified as a key metabolite associated with severity of renal failure and has the potential as a biomarker in assessing stages of chronic kidney diseases [48]. A recent report suggests that plasma 5-MTP has the potential to predict post-MI heart failure [50]. As 5-MTP could be a valuable therapeutic agent for various inflammation-mediated diseases, blood 5-MTP may be a useful theranostic biomarker.

## Conclusion

L-tryptophan, an essential amino acid for protein synthesis, is known to be converted in distinct cell types via distinct catabolic pathways to physiologically important metabolites such as kynurenines, serotonin and melatonin. Recent reports indicate that L-tryptophan is converted to a new class of factor i.e. 5-MTP with anti-inflammatory and protective actions. Vascular EC is a major site of 5-MTP synthesis. 5-MTP protects endothelial barrier function by preventing VE-cadherin degradation and defends against inflammation by controlling endothelial adhesion molecule expression, leukocyte transmigration and macrophage activation. Furthermore, 5-MTP protects endothelial survival and attenuates intimal hyperplasia in vascular injury by preventing vascular SMC phenotypic switch.

**Fig. 3:**
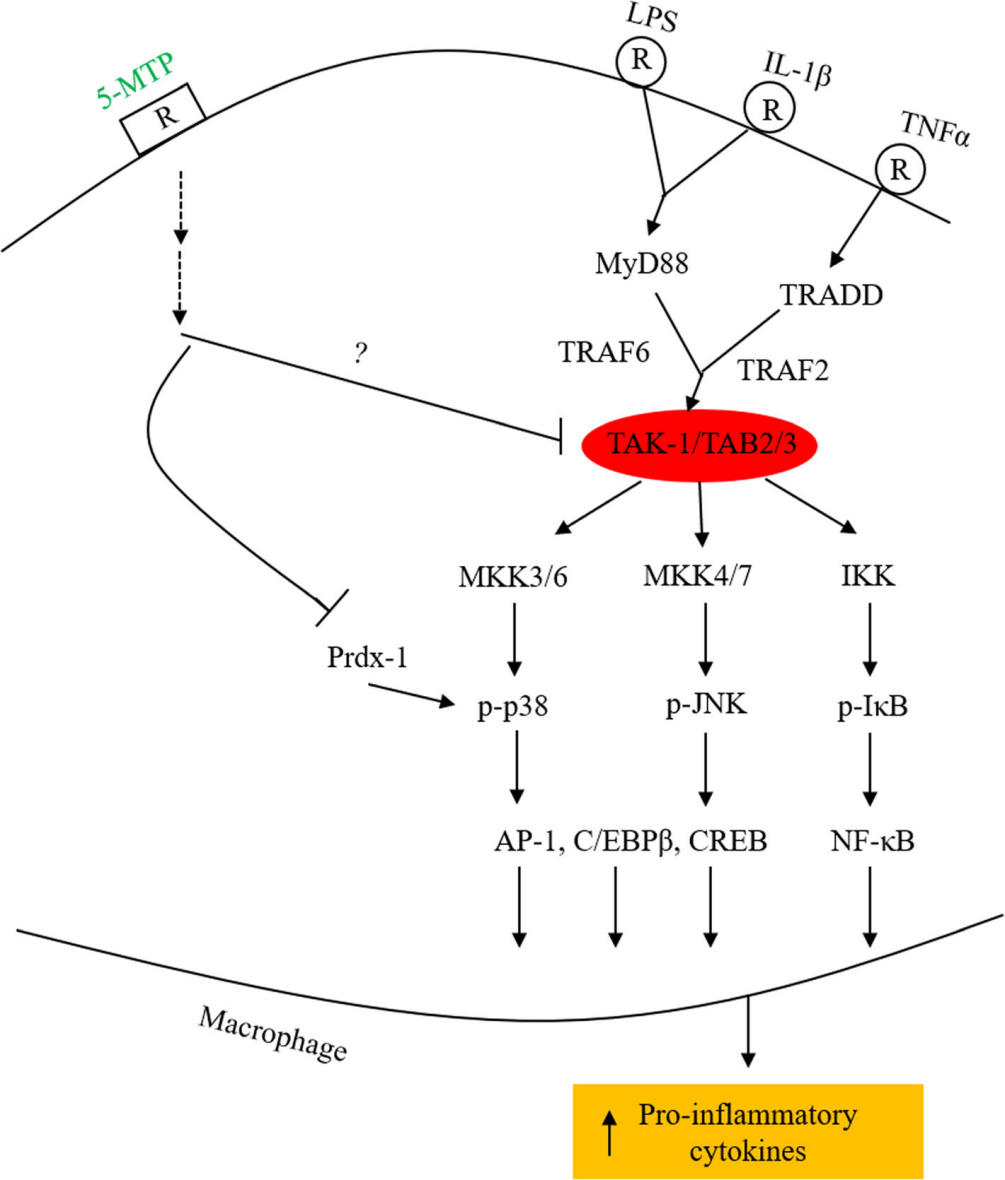
Schematic illustration of hypothetical mechanisms of 5-MTP actions. 5-MTP may exert anti-inflammation by inhibiting TAK-1

5-MTP protects endothelial VE-cadherin, blocks VSMC migration and proliferation and inhibits macrophage activation by targeting a common signaling kinase, i.e. p38 MAPK. p38 MAPK has been known to signal LPS- and cytokine-induced vascular leakage, intimal hyperplasia and macrophage-mediated systemic inflammation. Thus, by blocking p38 MAPK activation, 5-MTP protects endothelial integrity and defends against inflammation. 5-MTP inhibits sustained p38 MAPK activation by blocking interaction between Prdx-1 and p-p38 MAPK. However, it remains unclear how 5-MTP blocks p38 MAPK activation. As the action of 5-MTP may involve its interaction with a cell surface receptor [3], its effect on p38 MAPK may be mediated through a cross-talk between 5-MTP signaling and LPS- and cytokine-induced signaling pathways. The signaling pathways of LPS, IL-1β and TNFα in macrophages converge at TAK-1 which phosphorylates IKK as well as p38 MAPK, ERK and JNK, thereby activating multiple transactivators [51, 52]. Suppression of TAK-1 was reported to counteract the inflammatory endothelial phenotype induced by stress signals [53]. As 5-MTP blocks binding of p65 NFκB, C-Jun, C/EBPβ and CREB to COX-2 promoter in fibroblasts [40], it is possible that the 5-MTP signaling pathway may block the actions of LPS and cytokines by inhibiting TAK-1 (Fig. 3). This possible mechanism requires further investigation.

5-MTP possesses anti-inflammatory and anti-fibrotic actions and is effective in rescuing injured tissues and organs by controlling macrophage mediated systemic or local inflammation. 5-MTP has the potential as a lead compound to develop new classes of drugs for treating human renal and cardiac diseases due to ischemia-reperfusion injury. 5-MTP was reported to be effective in preventing fibrosis and chronic kidney disease in a murine renal injury model [49]. Our preliminary results reveal that 5-MTP administration following acute MI attenuates remodeling and fibrosis and preserves cardiac function. In addition, 5-MTP was reported to control “cytokine storm” and attenuate organ injury in murine sepsis models [3] and, thus, may serve as a lead compound to develop new anti-sepsis drugs. For intellectual property protection, we have synthesized a 5-MTP analog, i.e. 5-MTP methylester and evaluated its effects on LPS-induced macrophage activation and sepsis. The protective effect of 5-MTP methylester is comparable to that of 5-MTP (please see ref. [54] from a patent granted by U.S. patent office).

Serum or plasma 5-MTP levels are reduced in several inflammation-mediated human diseases such as sepsis, chronic kidney disease and post-MI heart failure. 5-MTP level was reported to be a potential biomarker for diagnosing and predicting severity of the disease. Blood 5-MTP may serve as a theranostic biomarker of those diseases.

## Data Availability

Not applicable.

## Abbreviations

EC: Endothelial cell
PGI_2_: Prostaglandin I_2_ or prostacyclin
NO: Nitric oxide
iNOS: Inducible NO synthase
COX-2: Cyclooxygenase-2
SMC: Smooth muscle cell
5-MTP: 5-methoxytryptophan
TPH-1: Tryptophan hydroxylase-1
5-HTP: 5-hydroxytryptophan
HIOMT: Hydroxyindole O-methyltransferase
ASMT: Acetylserotonin methyltransferase
HUVEC: Human umbilical vein endothelial cells
ER: Endoplasmic reticulum
VE-cadherin: Vascular endothelial cadherin
VEGF: Vascular endothelial growth factor
VEGFR 2: VEGF receptor 2
LPS: Lipopolysaccharide
MAPK: Mitogen-activated protein kinase
IL-1β: Interleukin-1β
TNFα: Tumor necrosis factor α
SRF: Serum response factor
MRTF: Myocardin-related transcription factor
TAK-1: Transforming growth factor β1-activated kinase-1
IKK: IκB kinase
MKK: MAPK kinase
IL-6: Interleukin 6
Prdx: Peroxiredoxins
UUO: Unilateral urethral obstruction
MI: Myocardial infarction
MyD88: Myeloid differentiation primary response 88
TRADD: TNFR-1 associated death domain
TRAF: TNF receptor associated factor
TAB: TAK binding protein
